# Responses of *In vitro*-Grown Plantlets (*Vitis vinifera*) to *Grapevine leafroll-Associated Virus-3* and PEG-Induced Drought Stress

**DOI:** 10.3389/fphys.2016.00203

**Published:** 2016-06-02

**Authors:** Zhen-Hua Cui, Wen-Lu Bi, Xin-Yi Hao, Yan Xu, Peng-Min Li, M. Andrew Walker, Qiao-Chun Wang

**Affiliations:** ^1^State Key Laboratory of Crop Stress Biology for Arid Areas, Key Laboratory of Genetic Improvement of Horticultural Crops of Northwest China, College of Horticulture, Northwest A&F UniversityYangling, China; ^2^Department of Viticulture and Enology, University of California, DavisDavis, CA, USA

**Keywords:** cell damage, drought, grapevine leafroll virus, physiological metabolism, plant hormones, *Vitis vinifera*

## Abstract

Stresses caused by viral diseases and drought have long threatened sustainable production of grapevine. These two stresses frequently occur simultaneously in many of grapevine growing regions of the world. We studied responses of *in vitro*-grown plantlets (*Vitis vinifera*) to *Grapevine leafroll associated virus-3* (GLRaV-3) and PEG-induced drought stress. Results showed that stress induced by either virus infection or drought had negative effects on vegetative growth, caused significant decreases and increases in total soluble protein and free proline, respectively, induced obvious cell membrane damage and cell death, and markedly increased accumulations of ${\text{O}}_{2}^{\cdot -}$ and H_2_O_2_. Co-stress by virus and drought had much severer effects than single stress on the said parameters. Virus infection alone did not cause significant alternations in activities of POD, ROS, and SOD, and contents of MDA, which, however, markedly increased in the plantlets when grown under single drought stress and co-stress by the virus and drought. Levels of ABA increased, while those of IAA decreased in the plantlets stressed by virus infection or drought. Simultaneous stresses by the virus and drought had co-effects on the levels of ABA and IAA. Up-regulation of expressions of ABA biosynthesis genes and down-regulation of expressions of IAA biosynthesis genes were responsible for the alternations of ABA and IAA levels induced by either the virus infection or drought stress and co-stress by them. Experimental strategies established in the present study using *in vitro* system facilitate investigations on ‘pure’ biotic and abiotic stress on plants. The results obtained here provide new insights into adverse effects of stress induced by virus and drought, in single and particularly their combination, on plants, and allow us to re-orientate agricultural managements toward sustainable development of the agriculture.

## Introduction

Stresses caused by abiotic and biotic factors have long threatened sustainable development of agricultural production. Drought, one of the greatest abiotic stresses, was reported to cause agricultural losses of 50 billion dollars between 1980 and 2012 in United States alone (Suzuki et al., 2014). It is estimated that more than 6% of the world's land and 30% of the world's irrigated areas also face salinity problems (UNESCO Water Portal, 2007). Viral disease, a biotic stress, causes serious economic losses to agricultural crops (Hadidi and Barba, 2011).

The influence of stress by either virus infection (Sampol et al., 2003; Moutinho-Pereira et al., 2012; Li et al., 2013; Cui et al., 2015) or drought (Zhu, 2002; Suzuki et al., 2014) has been well-demonstrated on plant development, growth, photosynthetic capability and various physiological metabolisms, eventually resulting in reduction of crop yield and quality. However, studies on their combining effects have been quite limited (Atkinson and Urwin, 2012; Prasch, 2013; Suzuki et al., 2014). Plants grown in nature are exposed simultaneously to abiotic and biotic stresses. Abiotic stress was shown to alter the ability of plants resist/tolerate pathogens (Atkinson and Urwin, 2012; Prasch, 2013). Similarly, biotic stress was found to alter resistance/tolerance of hosts to abiotic stress (Xu et al., 2008; Atkinson and Urwin, 2012; Prasch, 2013). Therefore, responses of plants to single stress differ from those to multiple stresses (Atkinson and Urwin, 2012; Suzuki et al., 2014), and the simultaneous occurrence of multiple stresses can cause complex plant responses (Atkinson and Urwin, 2012; Prasch, 2013; Suzuki et al., 2014). Thus, better understanding of effects of combining abiotic with biotic stress is of great significance and would allow us to orientate agricultural management strategies to ensure sustainable development of agricultural production.

Grapevine (*Vitis vinifera*) is an economically important fruit crop worldwide. Virus disease and drought stress are the two key factors limiting high yield and quality production of grapevine (Martelli, 2012). Co-occurrence of drought and virus infection is common in many of grapevine-growing regions. However, most of the previous studies on the said issues addressed single stress, while the co-stress by virus and drought has been far less studied.

The objective of the present study was, therefore, to study responses of grapevines (*Vitis vinifera*) to *Grapevine leafroll-associated virus-3* (GLRaV-3) and PEG-induced drought stress using *in vitro* culture system. The overall goal was to better understand the effect of abiotic and biotic stress, in single and particularly in combination, on growth, physiological metabolic processes and cell damage, thus providing valuable data upon which agricultural management strategies could be adjusted to allow more sustainable viticultural production.

## Materials and methods

### Maintenance of *In vitro* healthy and GLRaV-3 infected stock plantlets and establishment of PEG-induced drought stress

*Vitis vinifera* L. ‘Cabernet Sauvignon’, a major red wine cultivar grown worldwide and susceptible to GLRaV-3, was used in this study. *In vitro* shoots suspected of GLRaV-3 infection were established *in vitro*, according to Cui et al. (2015). After 6 months of *in vitro* establishment, the suspected shoots were screened again by RT-PCR for their virus status including GVA, GVB, GRSPaV, GFkV, GFLV, and ArMV, which are among the grapevine viruses reported in China (Ren et al., 2013). Samples showing a positive response only to GLRaV-3 were maintained and others discarded. The GLRaV-3 infected shoots (scions) were micrografted on to the healthy *in vitro* shoots (rootstocks). Micrografts developed into plantlets after 3 months of micrografting. GLRaV-3 was confirmed by RT-PCR in the micrografted rootstocks (Cui et al., 2015) and shoot segments were excised from the virus-infected rootstocks were proliferated to establish the diseased *in vitro stock* shoots. Thus, the healthy and diseased *in vitro* stock shoots were produced from the same mother plants. Both the healthy and virus-infected the cultures were maintained on a basal medium (BM) containing half-strength Murashige and Skoog (1962) medium (MS) with 30 g l^−1^ sucrose and 7 g l^−1^ agar. The pH of the medium was adjusted to 5.8 prior to autoclaving at 121°C for 20 min. The cultures were maintained at a constant temperature of 24 ± 2°C under a 16-h photoperiod with a light intensity of 50 μmol s^−1^ m^−2^ provided by cool-white fluorescent tubes. Subculture was done once every 6 weeks.

Shoot segments (1.5–2.0 cm in length) with 2 fully-opened leaves were excised from *in vitro* 6-week-old healthy and virus-infected stock plantlets, respectively, and cultured on BM containing 0, 2, or 4% (w/v) of polyethylene glycol (PEG) 8000, according to Cui et al. (2015), under the same cultural conditions as used for *in vitro* stock plantlets. Water potentials of medium containing 2 and 4% PEG were −2.4 MPa and −2.7 MPa, respectively, as calculated by Michel (1983). Unless stated otherwise, samples were taken after 4 weeks of culture and used for the following experiments.

### Vegetative growth of *In vitro* GLRaV-3 infected plantlets with and without drought stress

Vegetative growth including time required for axillary bud elongation, shoot length, number of roots, length of the longest root, and fresh weight (FW) and dry weight (DW) of shoots and roots were measured after 6 weeks of culture. Axillary bud elongation was defined when ≥50% of shoots showed bud elongation.

### Analysis of total soluble protein

Shoots with leaves of (0.5 g FW) were extracted with 3 ml of 50 mM phosphate buffer (pH 7.0), followed by centrifugation at 10,000 rpm for 10 min. The supernatant was collected and absorbance was recorded at 595 nm in a spectrophotometer (Thermo Multiskan MK3, USA) with bovine serum albumin (BSA) as a standard, according to Bradford (1976).

### Analysis of free proline

Free proline content was determined according to the method of Bates et al. (1973). Shoots with leaves (0.5 g FW) were homogenized with 10 ml of 3% (w/v) sulfosalicylic acid. The homogenate was centrifuged at 3000 rpm for 20 min. The supernatant was treated with acid ninhydrin in boiling water for 1 h. The reaction was terminated in a water bath at a room temperature for 10 min. The reaction mixture was extracted with 4 ml of toluene and vortexed for 15 s. The absorbance was determined at 520 nm in a spectrophotometer (Thermo Multiskan MK3, USA) using L-proline as a standard.

### Analysis of cell membrane damage (CMD) and cell death

Roots (0.5 g FW) were used for analysis of cell membrane damage (CMD) by measuring relative electrolytic leakage (EL), according to the method of Sullivan (1972). Cell death was detected by Evan's blue staining method (Gaff and Okong'O-Ogola, 1971). Samples were immersed in 0.1% Evan's blue solution for 30 min, and then washed with distilled water for 3 times to stop the color reaction. Staining reaction was photographed using a digital camera (PowerShot G9, Canon, Japan).

### ${\text{O}}_{2}^{\cdot -}$ and H_2_O_2_ localization *In situ*

Fresh leaves were used for ${\text{O}}_{2}^{\cdot -}$ and H_2_O_2_ localization *in situ*, according to the method of Romero-Puertas et al. (2004), with some modifications. For ${\text{O}}_{2}^{\cdot -}$ localization, leaves were immersed in 0.1% solution of nitroblue tetrazolium (NBT) in 10 mM K-phosphate buffer (pH 7.6), vacuum-infiltrated for 30 min and illuminated for 2 h, followed by bleaching in 95% boiling ethanol for 5 min. For H_2_O_2_ localization, leaves were immersed in a 0.1% filtered solution of 3, 3′-diaminobenzidine (DAB) in 10 mM MES buffer (pH 5), vacuum-infiltrated for 30 min and then incubated at room temperature for 4 h in the dark, followed by bleached in 95% boiling ethanol for 5 min. Photographs were captured by a digital camera (PowerShot G9, Canon, Japan).

### Analysis of antioxidant enzyme activities

The activities of superoxide dismutase (SOD), peroxidase (POD), catalase (CAT) were measured, as described by Li et al. (2011). Fresh leaves (0.5 g FW) were harvested from 2-week-old plantlets grown at 0 and 4% PEG, ground in liquid nitrogen and extracted with following extraction media: 100 mM potassium phosphate buffer (pH 7.8) containing 0.1 mM EDTA, 1% (w/v) PVP and 0.1% (v/v) Triton x100. The extracts were centrifuged at 10,000 rpm for 15 min at 4°C. The supernatants were collected and used for the enzyme activity assays.

The SOD activity was determined by measuring its ability to inhibit photochemical reduction of nitrobluetetrazolium (NBT). The reaction mixture (3 ml) contained 0.3 ml each of 20 μM riboflavin, 150 mM l-methionine, 600 μM NBT and 0.1 ml extract, and performed under irradiance of 170 μmol photons m^−2^ s^−1^ provided by white fluorescent lamps. The absorbance was determined at 560 nm. The extract volume causing 50% inhibition of NBT reduction was taken as one unit of activity. The POD activity was measured in the reaction mixture (3 ml) containing 50 mM phosphate buffer (pH 7.0), 0.2 mM guaiacol, 10 mM H_2_O_2_. The reaction was initiated by adding 200 μl of enzyme extract to the reaction mixture. The oxidation of guaiacol was measured upon an increase in absorbance at 470 nm for 1 min. The CAT activity was determined by directly measuring the decomposition of H_2_O_2_ at 240 nm in a spectrophotometer (Thermo Multiskan MK3, USA). The reaction mixture (3 ml) contained 0.05 M Na phosphate buffer (pH 7.0) supplemented with 1 mM EDTA, H_2_O_2_ (3%) and 100 μl of enzyme extract.

### Analysis of methane dicarboxylic aldehyde (MDA)

The MDA content was determined according to Li et al. (2011). Briefly, leaves (1.0 g FW) taken from 2-week-old plantlets grown at 0 and 4% PEG were homogenized in 10 ml of 10% trichloroacetic acid, followed by centrifugation at 10000 rpmg for 10 min. After then, 2 ml of 0.6% thiobarbituric acid in 10% trichloroacetic acid were added to 2 ml of the supernatant. The mixture was heated in boiling water for 15 min, and then quickly cooled in an ice bath. After centrifugation at 10,000 rpm for 10 min, the absorbance of the supernatant was recorded at 450 nm, 532 nm and 600 nm in a spectrophotometer (Thermo Multiskan MK3, USA). The MDA concentration was calculated using the following formula: 6.45(OD 532−OD 600)−0.56OD450.

### Analysis of endogenous hormones

Contents of endogenous ABA and IAA were analyzed in the *in vitro* plantlets grown at 0 and 4% PEG. Shoots with leaves were taken from 1-week-old plantlets and divided into two groups. One group was used for analysis of ABA and IAA, and the other for measurement of transcription level of ABA and IAA biosynthetic genes, as described below. Samples were frozen in liquid nitrogen and stored at −80°C until usage. Abscisic acid (ABA) and indoleactic acid (IAA) were provided by Sigma (St. Louis, MO, USA). At the beginning of extraction, 1000 Bq of each ABA and IAA, accordingly, were added to monitor the losses during purification. The extraction and purification of ABA and IAA were conducted, according to by Dobrev et al. (2005). The purified extract solution was transferred into 2-ml centrifuge tubes containing 0.3 g polyvinylpolypyrolidone (PVPP) and then kept at −20°C for the measurements of ABA and IAA in water breeze HPLC system (Waters 2489 UV/Visible Detector), using wavelength at 254 nm, velocity at 0.7 ml min^−1^ and sample quantity of 10 μm and column temperature at 30°C. The dried fraction containing ABA and IAA were, respectively, injected into the HPLC system for measurements, as described by Li et al. (2013).

### Analysis of transcription level of ABA and IAA biosynthetic genes

The transcription levels of several major genes responsible for biosynthesis of ABA and IAA were analyzed in the *in vitro* plantlets grown at 0 and 4% PEG at different time durations. RNA was isolated from shoots with leaves (1 g FW), as described by Davies and Robinson (1996). RNA quality was evaluated by optical density (OD) value (1.9–2.1) at 260/280 nm. After removal of DNA using DNA Eraser (Takara, Japan), cDNA was synthesized using the reagent kits (RR047A, Takara, Japan) according to the manufacture's instructions. All the candidate primers and reference genes of ABA and IAA biosynthesis pathway were selected according to the existing studies (Table 1). The reagent kits (RR047A, Takara) were used for qPCR reaction (IQ5, BIO-RAD, USA) in a 25 μl reaction mixture containing 12.5 μl of 2 × SYBR Green I Master Mix, 1 μl of each primer (0.4 μM), 2 μl of cDNA (100 ng) and 8.5 μl of RNase-free water according to the kits instructions. The following program was used: an initial denaturation step at 94°C for 30 s, 45 cycles at 94°C for 10 s, 56°C for 10 s and 72°C for 30 s. A melting curve analysis was carried out over the range 65–97°C to verify the specificity of amplicons. Two controls (no-RT and no-template) were included in the designs. Transcript levels of each gene were normalized according to the reference gene, using the 2-(-Delta Delta C (T) method (Livak and Schmittgen, 2001).

**Table 1:** **Primers used for qRT-PCR analysis of ABA and IAA in ***in vitro*** plantlets of grape ‘Cabernet Sauvignon’**.

**Primer names**	**References**	**Sequence 5′-3′(forward/reverse)**	PN40024 12X V1 ID^*^	Coordinates^*^
VvNCED1	Wheeler et al., 2009	GAGACCCCAACTCTGGCAGG/ AAGGTGCCGTGGAATCCATAG	VIT_19s0093g00550	chr19:17645348.17647649
VvNCED2	Wheeler et al., 2009	AGTTCCATACGGGTTTCATGGG/ CCATTTTCCAAATCCAGGGTGT	VIT_10s0003g03750	chr10:6374432.6376728
VvZEP	Wheeler et al., 2009	TACCGGGTATTTTTGGGACA/ CTTCTTCATCCGTGGCAAGT	VIT_07s0031g00620	chr7:16795707.16804559
VvTAR2	Böttcher et al., 2013	CAGCAATGAAGCATATTGAAGG/ GAGTGAGAGCACCAGGAAATG	VIT_17s0000g08990	chr17:10559797.10565113
VvTAR3	Böttcher et al., 2013	CCCAAGATGACT TTGATATGCTG/ TGATCAACTGATTGTTGATTCCACT	VIT_18s0157g00090	chr18:18858918.18864125
VvTAR4	Böttcher et al., 2013	CAGCCTCATCAAGACCCAAGAT/ TGACGGTTGATTTCATTCTTCG	VIT_18s0157g00170	chr18:18980507.18985732
VvYUC1	Böttcher et al., 2013	CAGGAAACTGTCGCAATAGTGG/ CAAGAACTATGTTGGGTATTGAGAGG	VIT_07s0104g01250	chr7:2276803.2279543
VvACTIN2	Böttcher et al., 2011	GCACCCTTCG CACGATATGA/ TGACGCAAGGCAAGGACTGA	VIT_04s0044g00580	chr4:21427077.21431176

* Means V1 annotations obtained from CRIBI (http://genomes.cribi.unipd.it/grape/) by a BLAST search of the gene sequence provided by NCBI (http://www.ncbi.nlm.nih.gov).

### Experimental design and data analysis

Since some of the leaves at the basal part of the diseased shoots showed GLRaV-3 symptoms when grown under PEG-induced stress (Cui et al., 2015), symptomless leaves were used in all experiments. The experiments determining effects of virus and drought stress on vegetative growth of *in vitro* plantlets were designed as a completely randomized design. Ten samples were included in each of three replicates and the whole experiment was repeated at least twice. In all experiments measuring contents of total soluble protein, proline and MDA, activities of SOD, POD and CAT, endogenous hormones and transcript levels of ABA and IAA biosynthetic genes, each treatment contained five biological replicates and repeated three times in each experiment. Statistical analysis of the data was performed with the SPSS-19 for Windows statistics software package. Significant differences among means were calculated by the least significant difference (LSD) at *P* ≤ 0.05. Two-way ANOVA, including GLRaV-3 infection and drought as factors, was performed to analyze the combined effects of these two factors on some selected parameters. Two-way MANOVA was performed for the vegetative growth. Significant differences were analyzed at *P* ≤ 0.05 and *P* ≤ 0.01, respectively.

## Results

### Vegetative growth and root formation

**Figure 1:**
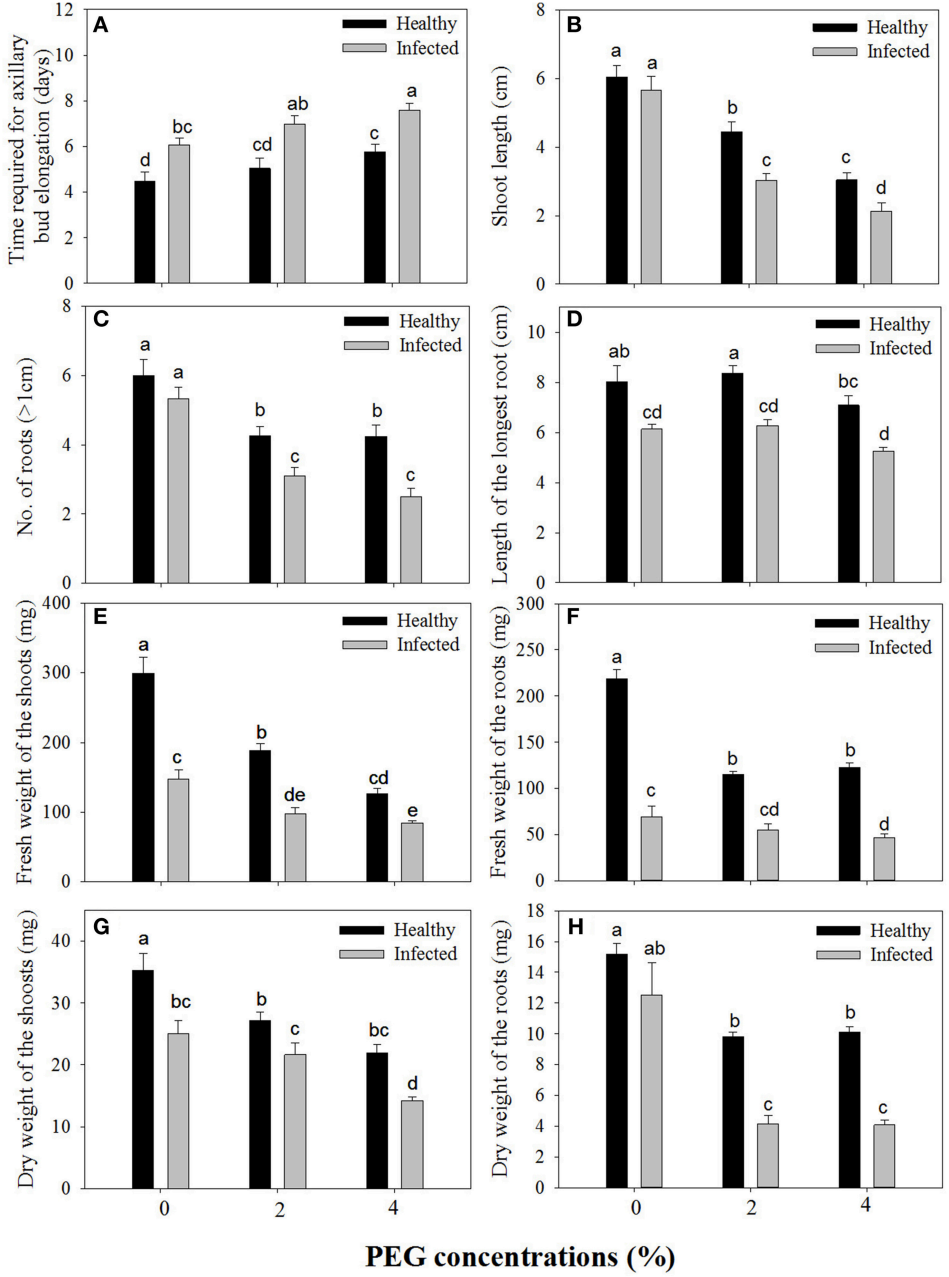
**Effects of GLRaV-3 infection and drought stress on shoot growth and root formation of ***in vitro*** plantlets of grape ‘Cabernet Sauvignon’**. **(A)** Time required for axillary bud elongation. **(B)** Shoot length. **(C)** Number of roots. **(D)** Length of the longest root. **(E)** Fresh weight of the shoots. **(F)** Fresh weight of the roots. **(G)** Dry weight of the shoots. **(H)** Dry weight of the roots. Data were presented as means ± SE and with different letters within the same parameter are significantly different at *P* ≤ 0.05.

Without PEG stress, time duration required for axillary bud elongation was much shorter in the healthy shoots (4.5 days) than in GLRaV-3 infected ones (6.1 days; Figure 1A). No significant differences were found in this parameter in the healthy shoots grown at 0% and 2% PEG, but PEG at 4% considerably delayed the time duration for axillary bud elongation (5.8 days). A similar pattern in axillary bud elongation was found in the diseased shoots grown at 2–4% PEG, but the negative effect was much stronger (Figure 1A). Shoot length was similar between the healthy and diseased shoots without PEG stress (Figure 1B). In the healthy shoots, shoot length markedly decreased as PEG concentrations increased from 0 to 4% (Figure 1B). This inhibitory effect exerted by PEG stress was much stronger on the infected shoots than on the healthy shoots (Figure 1B). When grown at 0% PEG, the healthy and virus infected shoots produced a similar number of roots (Figure 1C). Number of roots significantly decreased in the healthy shoots when grown at 2–4% PEG (Figure 1C). Negative effects of PEG concentrations on the number of roots were much pronounced in the infected shoots than in the healthy ones (Figure 1C). The healthy shoots without PEG stress produced greater length of the longest roots than the virus infected ones (Figure 1D). Length of the longest roots was similar in the healthy shoots grown at 0 and 2% PEG (Figure 1D), but 4% PEG resulted in much shorter length of the longest roots (Figure 1D). Co-stress by the virus and 4% PEG resulted in the shortest length of roots (Figure 1D). Without PEG stress, fresh weight (FW) of shoots was significantly greater in the healthy shoots than that of the virus infected ones (Figure 1E), and markedly reduced in the healthy shoots as PEG concentrations increased from 0 to 4%. Influences exerted by co-stress of the virus and drought on FW of roots were similar to those of FW of shoots (Figure 1F). Without PEG, the dry weight (DW) of the healthy shoots was greater than that of the virus infected shoots (Figure 1G), and it decreased when grown at 2–4% PEG. The DW of the infected shoots also decreased as PEG concentrations increased from 2 to 4%. There was no significant difference in DW of roots between the healthy and diseased shoots without PEG stress (Figure 1H). The DW of roots in both the healthy and infected shoots considerably reduced as PEG concentrations elevated from 0 to 2–4% (Figure 1H). Two-way MANOVA of all data on vegetative growth showed that single GLRaV-3 infection and PEG-induced drought, singly, and GLRaV-3 in combination with PEG all had significantly (*P* ≤ 0.01) negative effects on vegetative growth (Table 2).

**Table 2:** **Two-way MANOVA of effects of GLRaV-3 and PEG-induced drought, and their combing effects on all variables of vegetative growth of ***in vitro*** plantlets of grape ‘Cabernet Sauvignon’**.

**Parameters**	**Wilk's Lambda**	**F**	***p***
Virus	0.031	66.690	^**^
Drought	0.008	22.241	^**^
Virus-drought	0.003	37.855	^**^

** Significant difference at P ≤ 0.01.

### Content of total soluble protein and free proline

**Figure 2:**
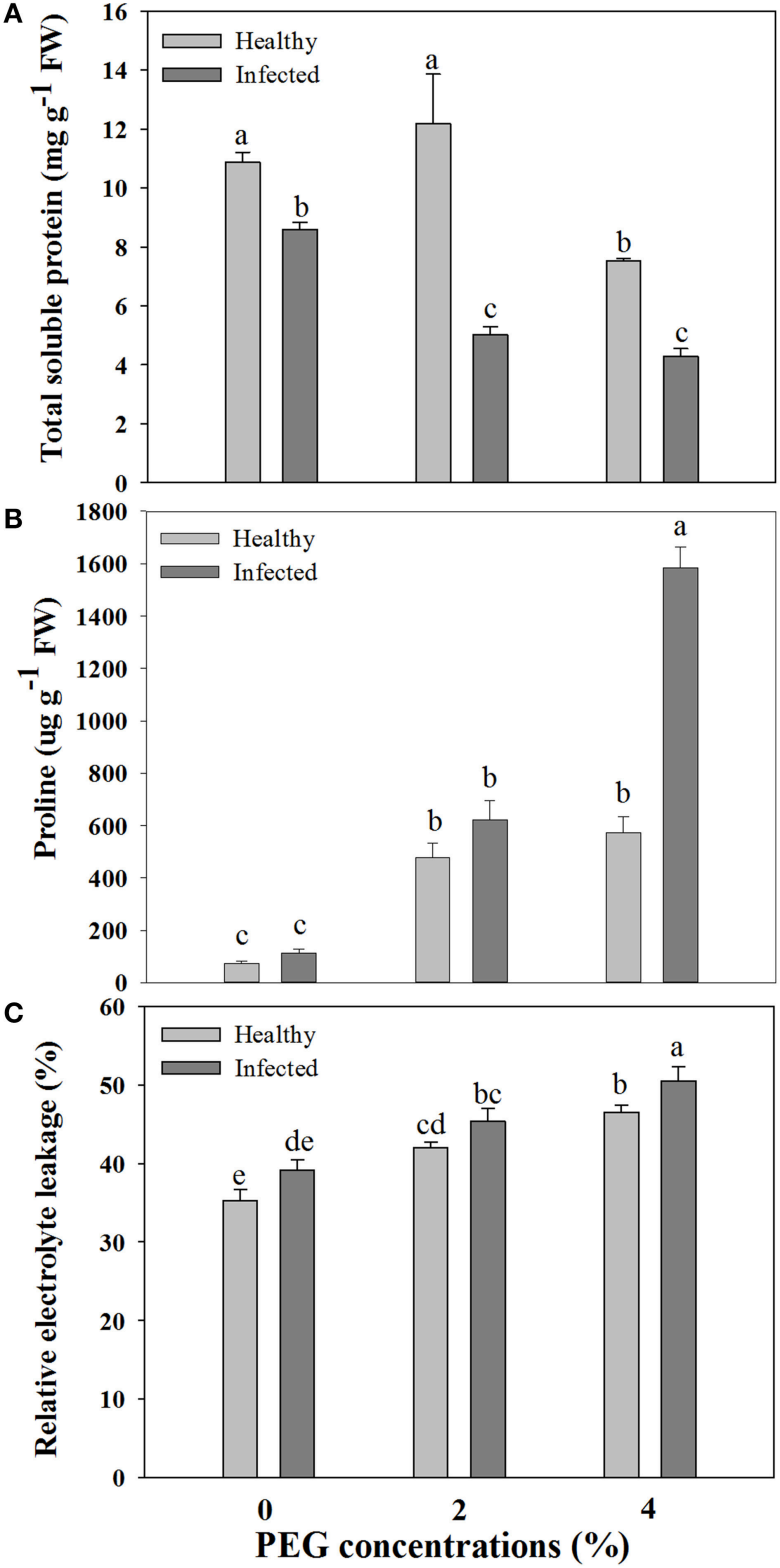
**Effects of GLRaV-3 infection and drought stress on contents of total soluble protein (A), proline (B), and relative electrolyte leakage (C) of ***in vitro*** plantlets of grape ‘Cabernet Sauvignon’**. Data are presented as means ± SE and with different letters are significantly different at *P* ≤ 0.05.

A significant difference was found in content of total soluble protein between the healthy and virus-infected shoots without PEG stress (Figure 2A). In the healthy shoots, marked reductions were found in content of total soluble protein only when grown at 4% PEG. In the infected shoots, PEG concentrations at 2–4% caused considerable decreases in contents of total soluble protein (Figure 2A). Content of free proline was similar in the healthy and diseased shoots without PEG stress (Figure 2B). In the healthy shoots, PEG at 2–4% significantly increased accumulations of free proline (Figure 2B). Levels of free proline considerably increased in the virus-infected shoots as PEG concentrations increased from 0 to 4% (Figure 2B). Two-way ANOVA showed that virus infection and PEG stress, and virus infection in combination with PEG stress had significant negative effects on contents of both total soluble protein and free proline (Table 3).

**Table 3:** **Two-way ANOVA of effects of GLRaV-3 and drought, and their combining effects on indexes of in vitro plantlets shoots of grape ‘Cabernet Sauvignon’**.

**Contents of Influenced indexes**	**Virus**	**Drought**	**Virus-Drought**
Total soluble protein	^**^	^**^	^*^
Proline	^**^	^**^	^**^
SOD	^**^	^**^	^*^
POD	^**^	^**^	^**^
CAT	^**^	^**^	^**^
MDA	^**^	^**^	^**^
ABA	^**^	^**^	^**^
IAA	^**^	^**^	ns

** Significant difference with levels of P ≤ 0.01;

* Significant difference with levels of P ≤ 0.05; ns = no significant difference.

### Cell membrane damage and cell death

Relative electrolytic leakage (EL) was similar in the healthy and diseased shoots without any stress (Figure 2C). Stress by PEG concentrations at 2 and 4% produced increased EL comparing to 0% PEG in the healthy shoots. The similar pattern of EL was found in the diseased shoots grown under 2–4% PEG concentrations, but the negative effect was much more obvious.

**Figure 3:**
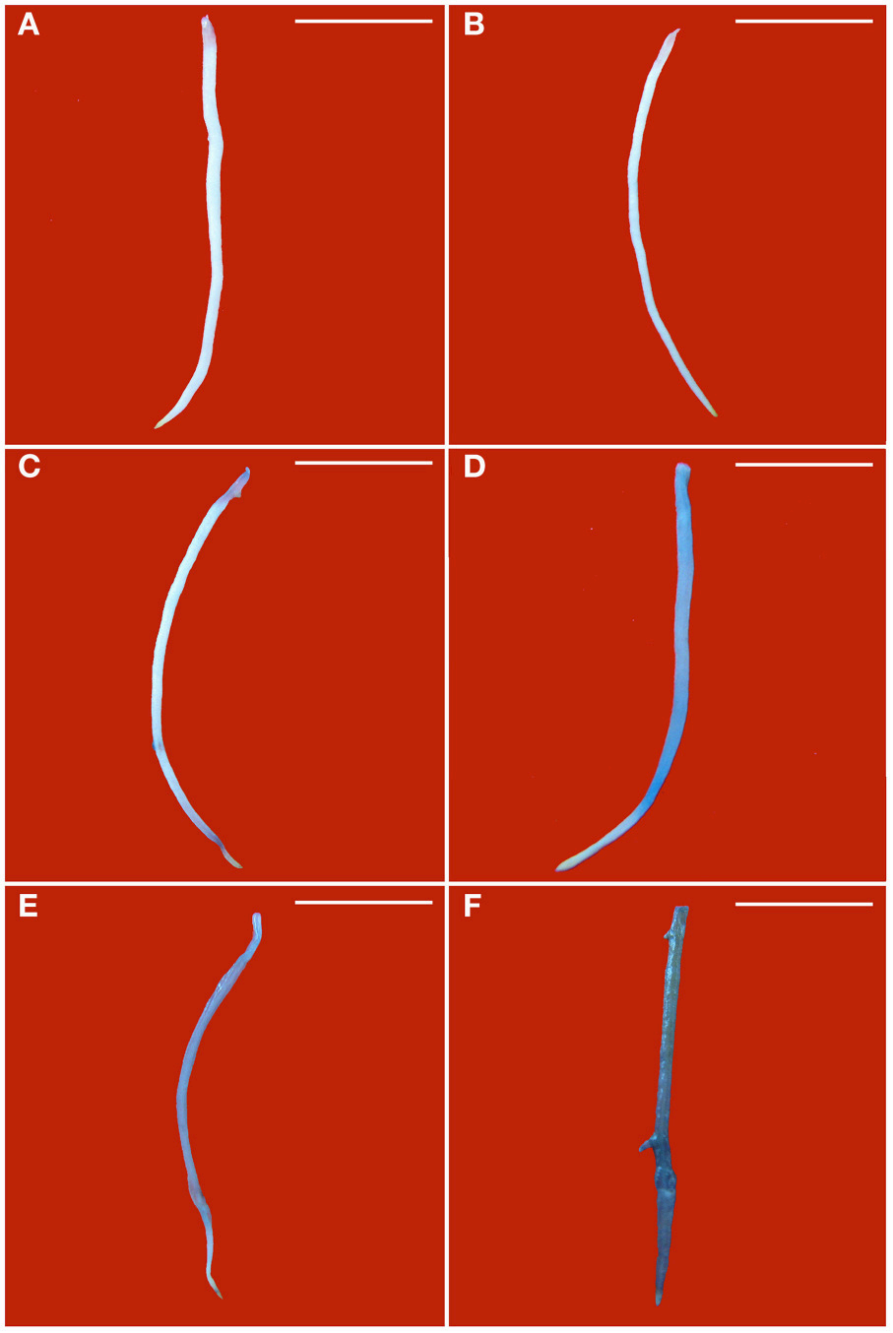
**Histochemical detection of cell death in roots of ***in vitro*** plantlets of grape ‘Cabernet Sauvignon’**. Cell death in the roots was revealed by the blue precipitates produced by staining with Evan's blue. Roots of the healthy shoots cultured at 0% **(A)**, 2% **(C)**, and 4% PEG **(E)**. Roots of GLRaV-3-infected shoots cultured at 0% **(B)**, 2% **(D)**, and 4% PEG **(F)**, Bar scale = 1 cm.

Blue color forms when dead cells are stained by Evan's blue solution. Without PEG stress, almost no blue color was seen in the healthy roots (Figure 3A), but some light blue color was observed in GLRaV-3 infected roots (Figure 3B). Density and areas of blue color formation increased with increasing PEG concentrations from 2 to 4% in both the healthy (Figures 3C,E) and virus-infected roots (Figures 3D,F). The virus-infected roots had a much stronger blue color and larger areas than the healthy roots that were treated by the same concentrations of PEG. When grown at 4% PEG, almost the whole roots infected by GLRaV-3 became blue (Figure 3F).

### ${\text{O}}_{2}^{\cdot -}$ and H_2_O_2_ localization *In situ*

**Figure 4:**
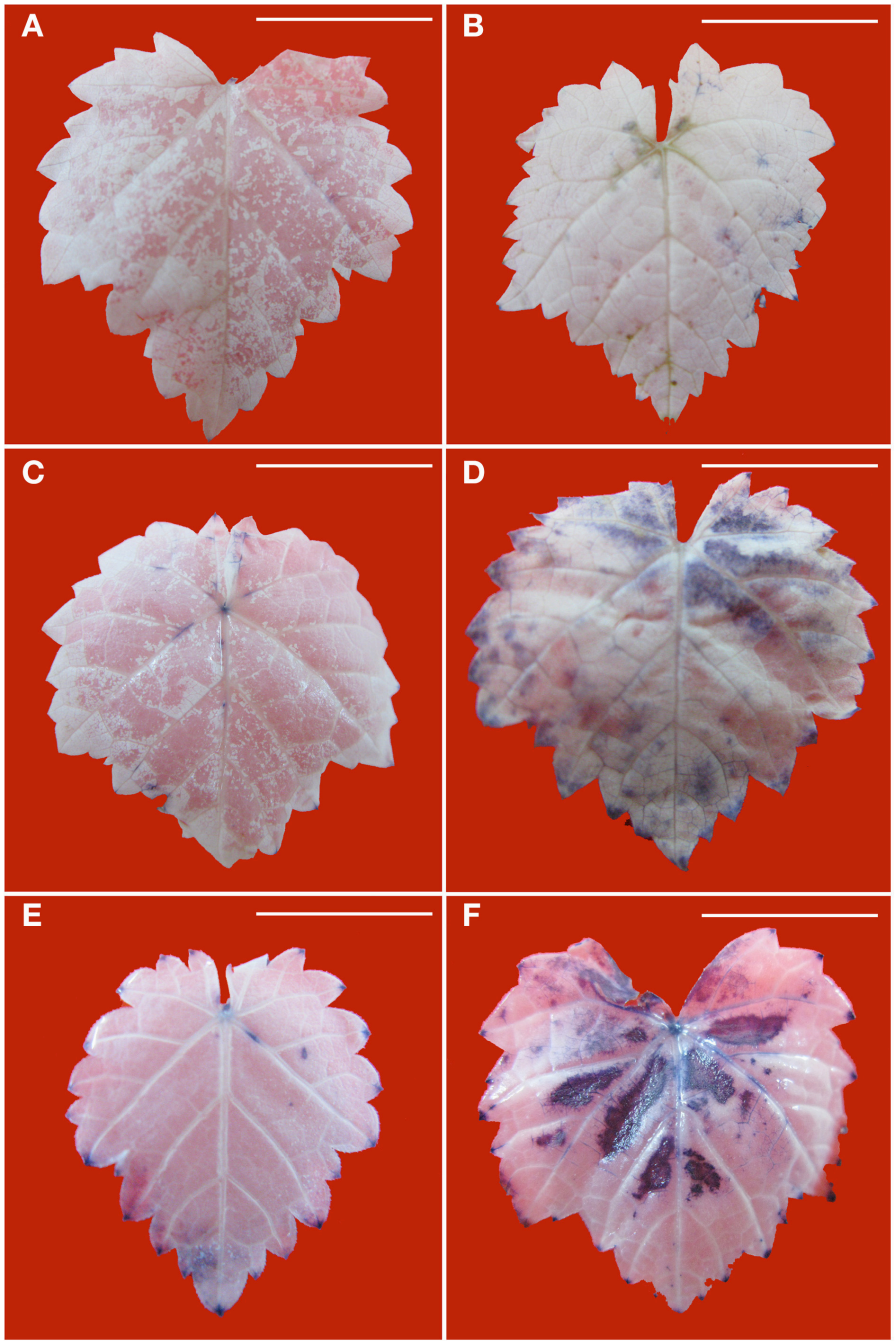
**Histochemical detection of ${\text{O}}_{2}^{\cdot -}$ in leaves of *in vitro* plantlets of grape ‘Cabernet Sauvignon’**. Deposits of ${\text{O}}_{2}^{\cdot -}$ were revealed by the blue formazan precipitates produced by staining with NBT. Leaves of the healthy *in vitro* shoots cultured at 0% **(A)**, 2% **(C)**, and 4% PEG **(E)**. Leaves of GLRaV-3-infected shoots cultured at 0% **(B)**, 2% **(D)**, and 4% PEG **(F)**. Bar scale = 1 cm.

**Figure 5:**
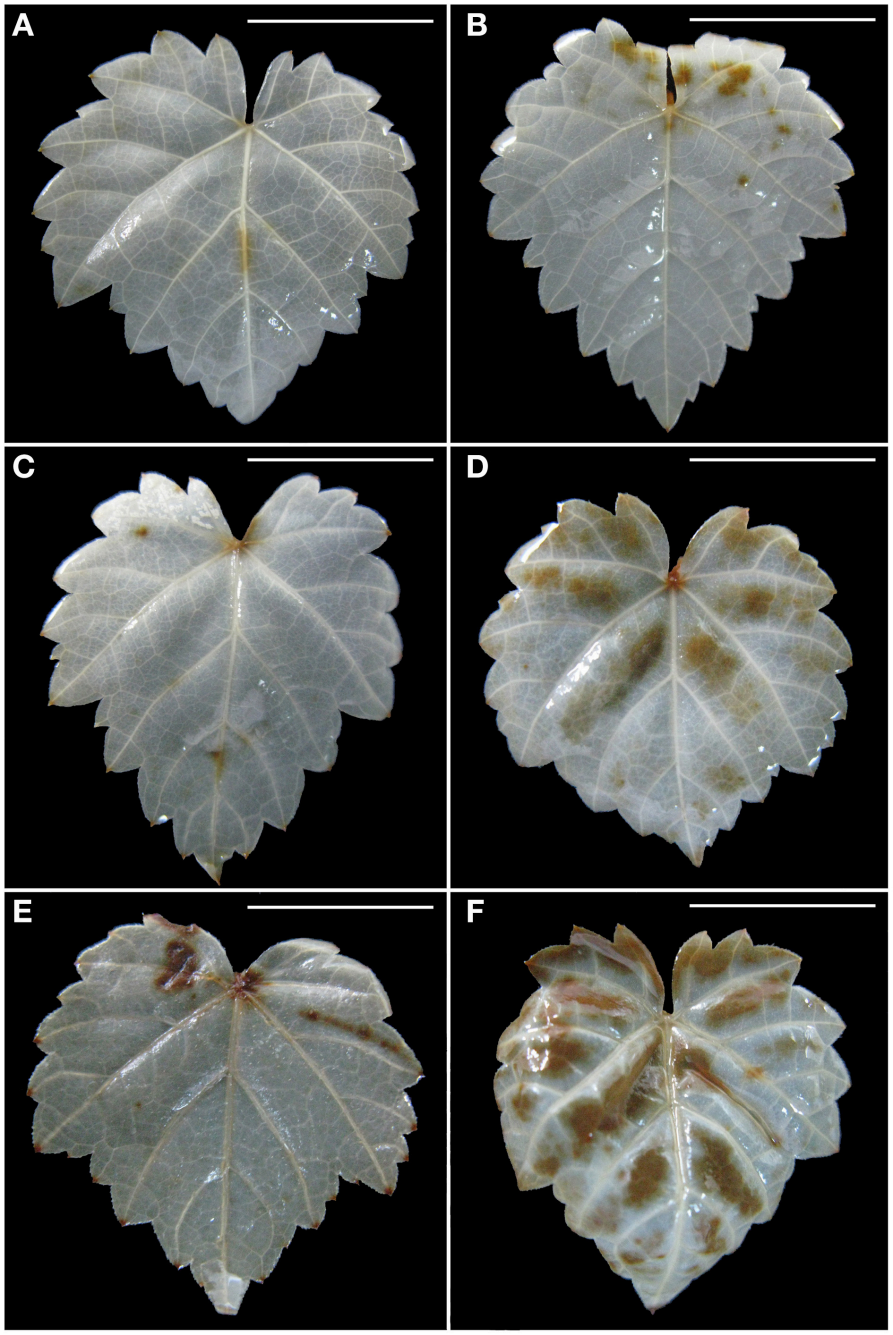
**Histochemical detection of H_**2**_**O**_**2**_ in leaves of ***in vitro*** plantlets of grape ‘Cabernet Sauvignon’**. H_2_O_2_ deposits were revealed by the brown precipitates produced by staining with DAB. Leaves of the healthy shoots cultured at 0% **(A)**, 2% **(C)**, and 4% PEG **(E)**. Leaves of LRaV-3-infected shoots cultured at 0% **(B)**, 2% **(D)**, and 4% PEG **(F)**. Bar scale = 1 cm.

Production of ${\text{O}}_{2}^{\cdot -}$ in the leaves is visualized with NBT staining to give rise to dark blue spots. Without PEG stress, ${\text{O}}_{2}^{\cdot -}$ accumulation was hardly detected in the healthy leaves (Figure 4A), but it was easily seen in the diseased leaves (Figure 4B). In the healthy leaves, ${\text{O}}_{2}^{\cdot -}$ accumulation considerably increased with an increase in PEG concentrations from 2% (Figure 4C) to 4% (Figure 4E). A similar pattern was found in the diseased leaves, but staining density and stained areas were much stronger (Figure 4D) and larger (Figure 4F) than in the corresponding healthy samples. Production of H_2_O_2_ in the leaves is visualized with DAB staining to give rise to brown spots. Without PEG, H_2_O_2_ accumulation in the virus-infected leaves (Figure 5B) was stronger than that in the healthy leaves (Figure 5A). Although, PEG stress at 2–4% markedly increased H_2_O_2_ accumulation in both the healthy and infected leaves, staining density and strained areas were much stronger and larger in the virus-infected leaves than in the healthy leaves that were stressed by the corresponding PEG concentrations (Figures 5C–F).

### Activities of antioxidant enzymes and content of MDA

**Figure 6:**
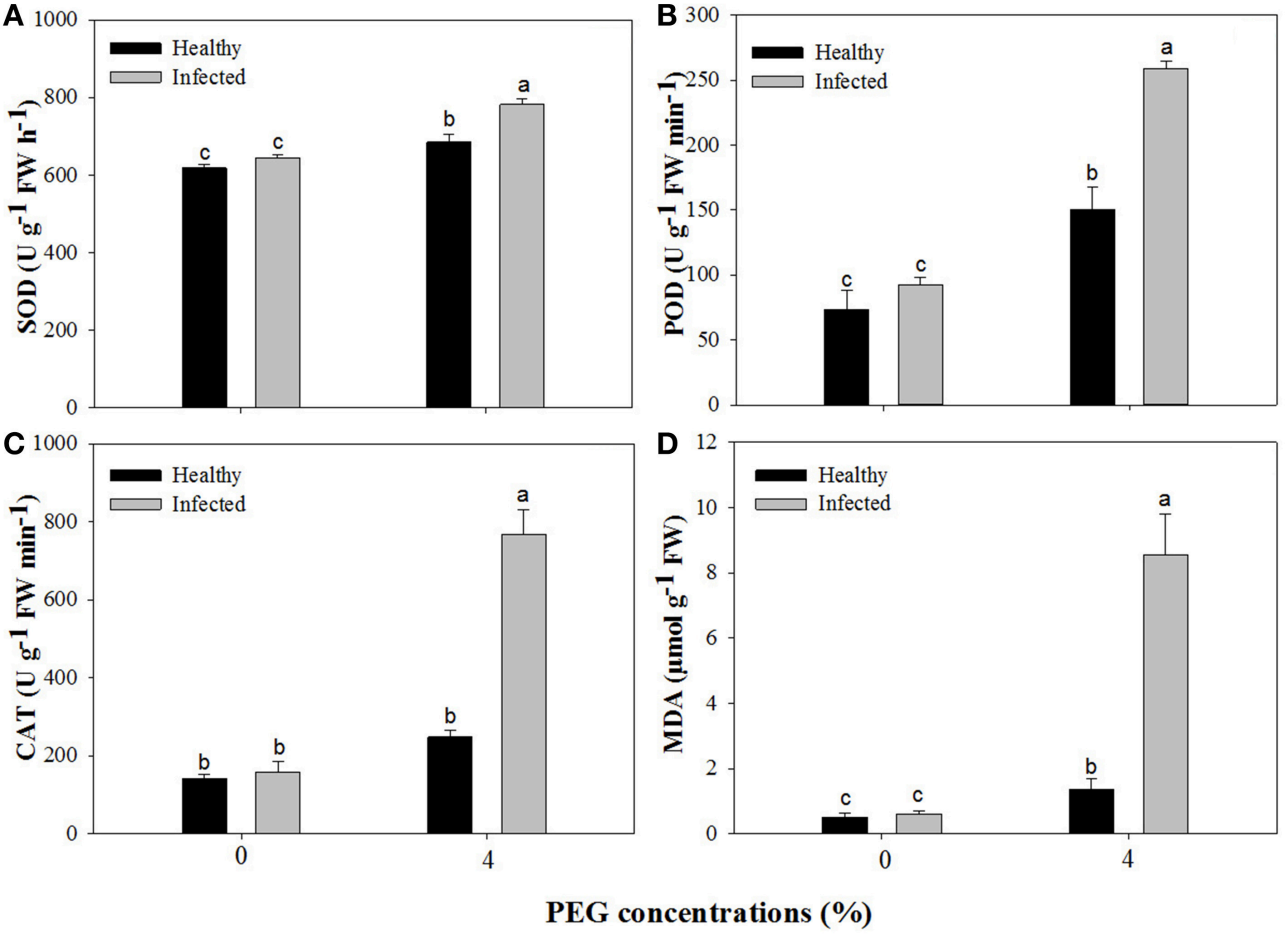
**Activities of superoxide dismutase (SOD, A), peroxidase (POD, B), catalase (CAT, C), and content of methane dicarboxylic aldehyde (MDA, D) of ***in vitro*** plantlets of grape ‘Cabernet Sauvignon’**. Data were presented as means ± SE and with different letters within the same parameter are significantly different at *P* ≤ 0.05.

Without PEG stress, no significant differences were found in the activities of SOD, POD, and CAT between the virus-infected and healthy plantlets (Figures 6A–C). For the healthy plantlets, 4% PEG resulted in significant increases in the activities of SOD and POD, although not in CAT (Figures 6A–C). For the diseased plantlets, the activities of SOD, POD, and CAT significantly increased when stressed by PEG 4%. No differences were found in MDA contents between the healthy and diseased plantlets without PEG stress (Figure 6D). MDA contents were found much higher both in the healthy and the diseased plantlets grown at 4% PEG, (Figure 6D). Two-way ANOVA showed that single stress by either the virus or PEG significantly (*P* ≤ 0.01) changed the activities of SOD, POD, and CAT, and increased MDA content (Table 3). Co-stress by the virus and PEG had significantly (*P* ≤ 0.01) negative effects on those of POD, CAT and MDA, as well as on SOD (*P* ≤ 0.05) (Table 3).

### Contents of ABA and IAA, and expression levels of ABA and IAA biosynthetic genes

**Figure 7:**
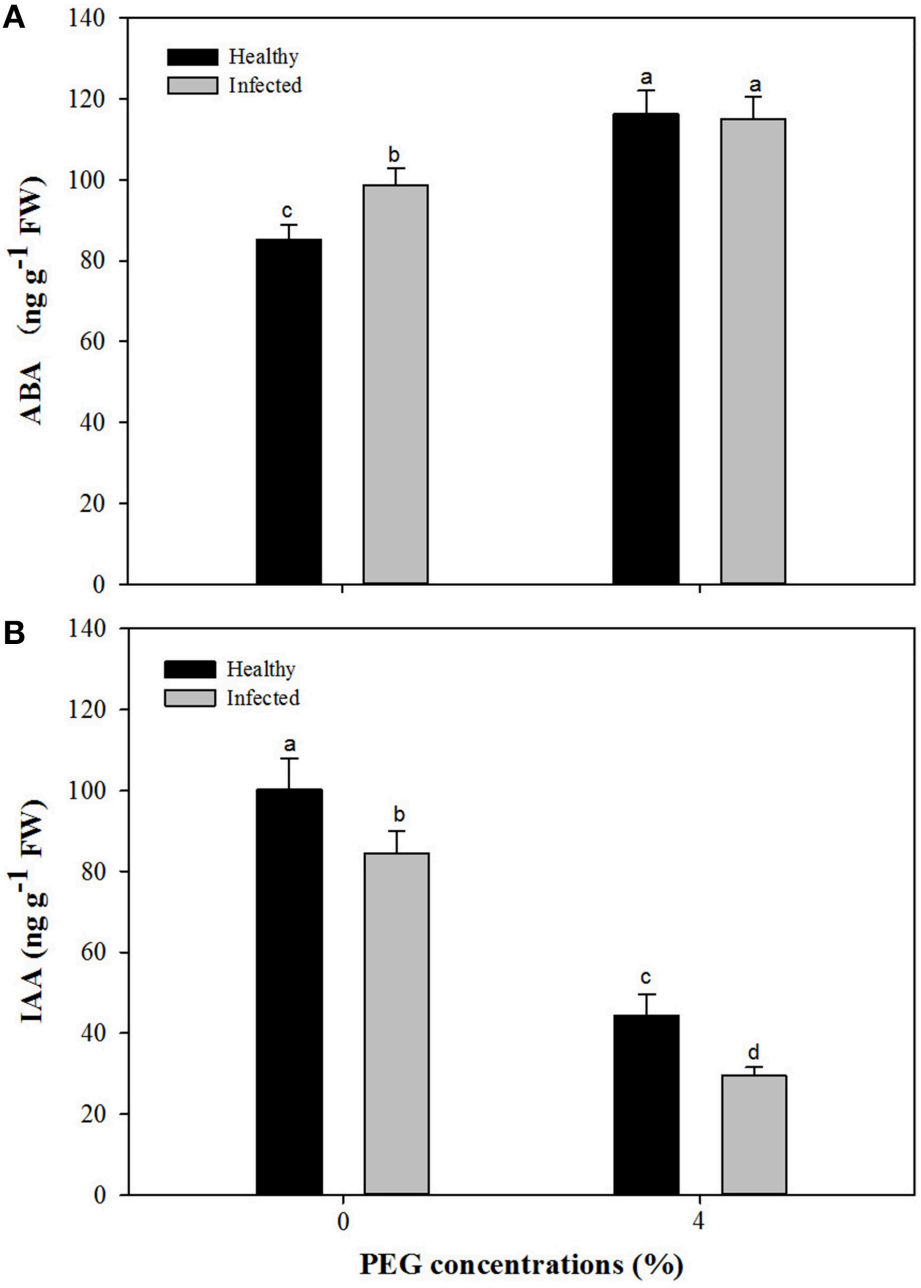
**Effects of GLRaV-3 infection and drought stress on content of ABA (A) and IAA (B) of ***in vitro*** plantlets of grape ‘Cabernet Sauvignon’**. Data were presented as means ± SE and with different letters within the same parameter are significantly different at *P* ≤ 0.05.

Without PEG stress, virus infection caused significant (*P* ≤ 0.05) increase and decrease in the content of ABA and IAA (Figures 7A,B), respectively. For the healthy plantlets, the content of ABA was significantly higher, while that of IAA was much lower, in the plantlets grown at 4% PEG than at 0% PEG (Figures 7A,B). Similar patterns of ABA and IAA levels were found in the virus-infected plantlets grown at 0 and 4% PEG (Figures 7A,B). Two-way ANOVA showed that PEG stress or the virus infection and their co-stress all significantly (*P* ≤ 0.01) increased ABA content; the virus infection or drought stress significantly (*P* ≤ 0.01) decreased IAA level, but there was no interaction between them on IAA level (Table 3).

**Figure 8:**
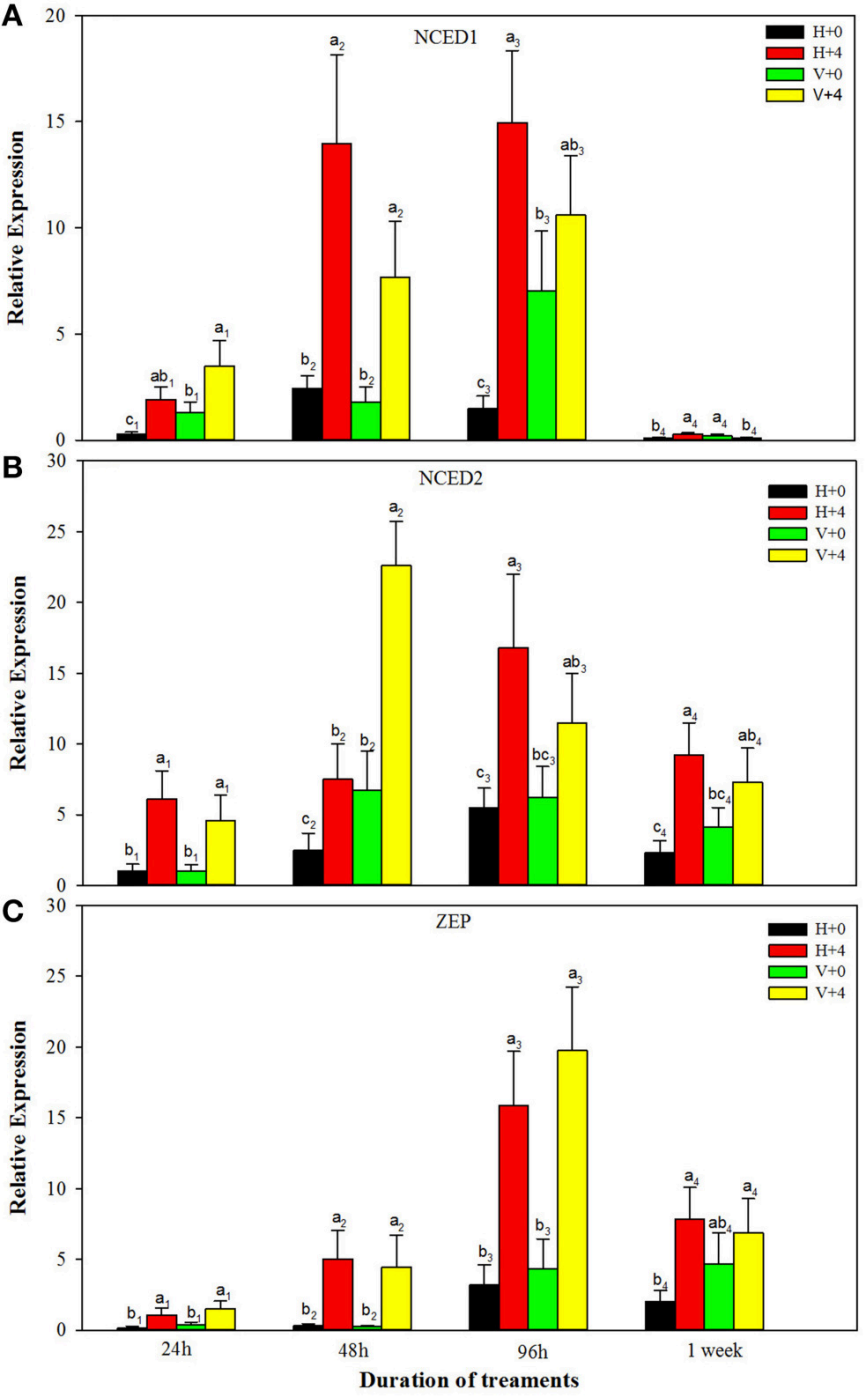
**Relative expressions of the ABA biosynthesis genes ***NCED1*** (A), ***NCED2*** (B), and ***ZEP*** (C) of ***in vitro*** plantlets of grape ‘Cabernet Sauvignon’ at different time points**. H+0 and H+4 = healthy plantlets with 0 and 4% PEG stress, respectively. V+0 and V+4 = virus-infected plants with 0 and 4% PEG stress, respectively. Data were presented as means ± SE and with different letters within the same time of analysis are significantly different at *P* ≤ 0.05.

**Figure 9:**
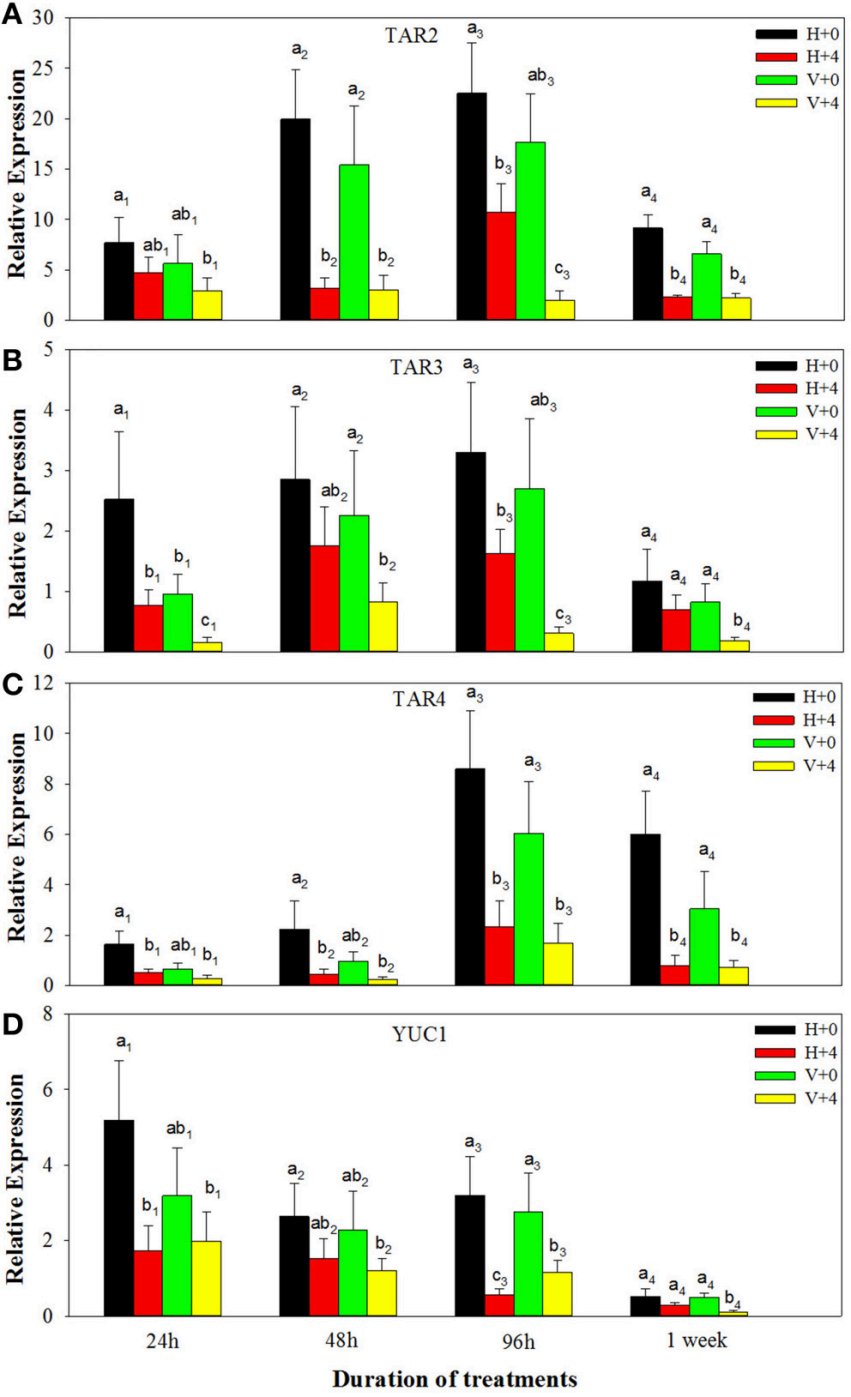
**Relative expressions of the IAA biosynthesis genes ***TAR2*** (A), ***TAR3*** (B), ***TAR4*** (C), and ***YUC1*** (D) of ***in vitro*** plantlets of grape ‘Cabernet Sauvignon’ at different time points**. H+0 and H+4 = healthy plants with 0 and 4% PEG stress, respectively. V+0 and V+4 = virus-infected plants with 0 and 4% PEG stress, respectively. Data were presented as means ± SE and with different letters within the same time of analysis are significantly different at *P* ≤ 0.05.

For ABA biosynthesis genes, without PEG stress, expression levels of NCED1 were relatively stable in the healthy plantlets during 1 week of culture, and peaked at 96 h of culture and then reduced in the virus infected plantlets (Figure 8A). Patterns of expression levels of NCED2 and ZEP were similar to those of NCED1 in the healthy plantlets (Figures 8B,C). In the diseased plantlets, expression levels of NCED2 started to increase at 48 h of culture and the increased levels maintained up to 1 week of culture (Figure 8B). Expression levels of ZEP were hardly detected until 48 h of culture, and relatively increased during 96 h and 1 week of culture (Figure 8C). PEG stress markedly induced expression levels of NCED1, NCED2, and ZE in both the healthy and diseased plantlets (Figures 8A–C). The highest expression levels appeared for NCED1 between 48 to 96 h in both the healthy and diseased plantlets (Figure 8A), for NCED2 at 48 and 96 h in the infected and healthy plantlets, respectively (Figure 8B), for ZEP at 96 h in the two types of plantlets (Figure 8C). For IAA biosynthesis genes, without PEG stress, higher expression levels of TAR2, TAR3, TAR4, and YUC1 were detected in the healthy than in the diseased plantlets during the whole culture time durations (Figures 9A–D). Expression levels of TAR2, TAR3, and TAR4 started to increase at 24 h, peaked at 96 h of culture and then decreased, while those of YUC1 peaked at 24 h and gradually decreased during 24 h to 1 weeks of culture (Figure 9D). PEG stress consistently reduced the expression levels of all the four genes in both the healthy and diseased plantlets during the whole culture durations, with more obviously reduced levels found in the diseased plantlets (Figures 9A–D).

## Discussion

Although, *in vitro* culture conditions largely differ from the field environments and responses of *in vitro* cultures to abiotic or biotic stress may differ from those of the field-grown plants in some cases (Suzuki et al., 2014), the former has been widely used for studies on the said subjects (Watanabe et al., 2000; Khristov et al., 2001; Christov et al., 2007; Lu et al., 2007; Lokhande et al., 2010, 2011; Faraloni et al., 2011; Li et al., 2013; Marković et al., 2014; Cui et al., 2015), mainly due to its advantages that can avoid other variables such as light, temperature and nutrients, and by attacks of other pathogens facing in the field, in addition to its effects of efficient, rapid and low cost, thus facilitating studies of ‘pure’ biotic or abiotic stress (Watanabe et al., 2000; Khristov et al., 2001; Christov et al., 2007; Lokhande et al., 2010, 2011; Li et al., 2013; Cui et al., 2015). A recent study of Faraloni et al. (2011) found a high accordance between the *in vitro* and *in vivo* chlorophyll fluorescence measurements in monitoring changes in photosynthetic activity in response to drought stress.

Effects of single stress by either virus or drought on plant growth have been well-documented, but co-stress by pathogens like virus and drought to plants has been far less studied. Working on the several grapevine cultivars, Marković et al. (2014) found that the healthy *in vitro*-grown shoots had much better shoot growth than the GLRaV-3 or other virus infected ones. Similar results have been reported in *in vitro* grapevine shoots infected by GLRaV-3 (Cui et al., 2015), *Grapevine leafroll* (Tanne et al., 1996), and *Grapevine fanleaf virus* (GFLV) (Abracheva et al., 1994). A number of examples of negative effects of virus infection on plant growth can be found in the publications of Watanabe et al. (2000), Li et al. (2013), and Cui et al. (2015). Reduced vegetative growth and slow rooting were observed in the i*n vitro* grapevine shoots stressed by 2–6% PEG (Dami and Hughes, 1997) and by 4% sorbitol or 2–4% mannitol (Tanne et al., 1996). Inhibitory effects exerted by drought or osmotic stress on vegetative growth were much stronger in the grapevine leafroll infected shoots than in the healthy ones (Chaves et al., 2007; Cui et al., 2015). All of these results were consistent with ours.

Virus infection or drought stress has been found to influence levels of soluble protein. An increased level of soluble protein was noted in grapevine infected with GFLV and GLRaV-3 (Sampol et al., 2003) or GLRV (Bertamini et al., 2004; Moutinho-Pereira et al., 2012), in banana infected with BBTV (Haq et al., 2012) and in potato infected with PLRV or PVY (Li et al., 2013). Similar results were also found in the present study. The decrease in synthesis of ribulose-1,5-bisphosphate (RuBP) carboxylase was found responsible for the marked reduction in the content of soluble proteins in the GLRV-infected samples (Bertamini et al., 2004). Decreased contents of total protein were observed in drought-stressed grapevine (Maroco et al., 2002) and other plants such as *Zea mays* (Hsiao, 1970), and *Gossypium hirsutum* (Parida et al., 2007). These data were again confirmed in our study. Drought stress caused marked changes in the protein synthesizing apparatus of plant tissue, thus reducing capacity for protein synthesis (Hsiao, 1970; Parida et al., 2007).

Our study found that drought stress and GLRaV-3 infection, singly or in combination, significantly increased proline contents. Increased proline contents by drought have been found in other plant species such as *Populus euphratic* (Watanabe et al., 2000), *Eucalyptus camaldulensis* (Woodward and Bennett, 2005) and *Capsicum annuum* (Fu et al., 2009), or by virus such as *Zucchini yellow mosaic virus* (ZYMV, Radwan et al., 2007). Proline accumulation generally is considered as a drought tolerance mechanism in plants (Chutia and Borah, 2012; Sun et al., 2013). Increased accumulation of proline was believed to be as an adaptive response to the stress, thus helping to maintain cell membrane integrity and protecting subcellular structures in drought-stressed plants (Chutia and Borah, 2012; Sun et al., 2013), and to activate a hypersensitive response (HR) to virus infection (Radwan et al., 2007).

Our results showed that GLRaV-3 infection did not significantly cause cell membrane damage and cell death, but drought, singly or in combination with the virus, markedly increased cell membrane damage and induced cell death, particularly when GLRaV-3 infected shoots were stressed by 4% PEG. Programmed cell death (PCD) has been widely observed in response of the plants to pathogenic infection (Greenberg and Yao, 2004). The HR in response to virus infection is a typical example of PCD (Greenberg and Yao, 2004). Increased cell membrane damage and cell death have been well established in the plants infected with various viruses such as *Tobacco mosaic virus* (TMV)-infected *Nicotiana tabacum* (del Pozo and Lam, 2003), *Groundnut bud necrosis virus* (GBNV)-infected *Vigna unguiculata* (Permar et al., 2014) and *Soybean mosaic virus* (SMV)-infected *Glycine max* (Zhang et al., 2014). The HR is typically induced upon virus infection as in interaction between host encoded resistance (R) proteins and pathogen encoded avirulence proteins and functions to limit virus replication and thereby its eventual movement inside the host (Padder, 2014). Increased cell membrane damage and induced cell death by drought stress has also been found in other plants including *Saccharum officinarum* (Patade et al., 2008), *Arabidopsis* (Duan et al., 2010), and *Cucumis sativus* (Zhang et al., 2013). Drought stress-induced root cell death was suggested to be an adaptive response to the stress (Duan et al., 2010). Expression of *BAX inhibitor-1* (*AtBI1*) increased in response to water stress in roots of *Arabidopsis*, indicating that *AtBI1* and the endoplasmic reticulum (ER) response pathway reduced water stress-induced PCD (Duan et al., 2010).

MDA, a marker for lipid peroxidation, is frequently used as an indicator for measurement of cellular membrane damage (Masia, 2003). There have been many studies on drought-induced MDA, but data on virus-induced MDA have been quite limited. Huang et al. (2008) and Li et al. (2011) found the MDA level markedly increased in the plants under drought, indicating the occurrence of damage to cell membranes. These data were consistent with ours. In our study, virus infection did not cause significant accumulation of MDA, while 4% PEG resulted in significant increase in MAD and the increased MAD was much obvious in plantlets co-stressed by drought and the virus, indicating extra injury to cell membranes by the interaction of drought stress and virus-infection.

Reactive oxygen species (ROS), particularly ${\text{O}}_{2}^{\cdot -}$ and H_2_O_2_, are subproducts responding to abiotic and biotic stress (Bolwell et al., 1999). A number of studies have shown that accumulation of ${\text{O}}_{2}^{\cdot -}$ and H_2_O_2_ considerably increased in plants infected by virus (Evans et al., 2006; Zhang et al., 2014) or stressed by drought (Duan et al., 2010; Gill and Tuteja, 2010; Sun et al., 2013; Noctor et al., 2014). In the present study, we found that stress by GLRaV-3 infection or drought increased the accumulation of ${\text{O}}_{2}^{\cdot -}$ and H_2_O_2_, and that accumulation was much greater when the *in vitro* shoots were stressed simultaneously by GLRaV-3 and PEG-induced drought. ROS has been shown to influence the expression of a number of genes and therefore manipulate various processes including growth, PCD, abiotic stress responses and pathogen defense (Gill and Tuteja, 2010). The production of ROS is the first response when plants are under biotic and abiotic stress (Gara et al., 2003; Evans et al., 2006), and as the stress continues, massive and prolonged ROS production, called an oxidative burst, occurs in the stressed cells (Gara et al., 2003). Accumulation of ROS in the virus-infected (Evans et al., 2006) or in abiotic-stressed plants may be associated with stress-induced oxidative bursts (Gara et al., 2003; Evans et al., 2006). Such oxidative bursts serve a number of protective functions activating defense responses of plants to biotic and abiotic stress (Gara et al., 2003).

Numerous studies have demonstrated that plants are able to protect against abiotic stress (Gill and Tuteja, 2010; Li et al., 2011) or biotic stress (Gonçalves et al., 2013; Kyseláková et al., 2013) by increasing the activities of antioxidant enzymes such as SOD, CAT, and POD. In the present study, we found that drought stress significantly increased the activities of SOD, POD, and CAT in both the healthy and diseased plantlets, but single virus infection increased slightly the activity of antioxidant enzymes. The increased activities of three antioxidant enzymes were much higher in the plantlets co-stressed by the drought and virus, indicating a strong need for ROS removal. Transgenic plants that showed increase in CAT or POD or SOD activity exhibited enhanced tolerance to drought or pathogen resistance (Gill and Tuteja, 2010). SOD was believed to be the first line of defense against the toxic effects of elevated levels of ROS caused by abiotic or biotic stress (Gill and Tuteja, 2010).

ABA generally inhibits plant growth and is thought to act as a messenger when plants were stressed by either drought or virus (Jameson and Clarke, 2002; Zhu, 2002). Increased accumulation in ABA in plants under drought stress has been well-documented (Zhu, 2002), whereas there existed only a few studies on virus-induced ABA and no studies on changes of ABA levels in plants co-stressed by drought and virus (Jameson and Clarke, 2002). The present study confirmed again grapevine *in vitro* plantlets responded to drought stress by increasing ABA level, and found the level of ABA was higher in the GLRaV-3 infected plantlets than in the healthy ones. Similar results were also reported in plants infected with other viruses (Fraser and Whenham, 1989; Wang et al., 2011; Alazem et al., 2014). The increased levels of ABA were associated with up-regulation of ABA biosynthesis genes including NCED1, NCED2, and ZEP, which were the major genes for ABA biosynthesis in grapevine (Wheeler et al., 2009). These findings supported the speculation of Alazem et al. (2014) that the induction of ABA and up-regulation of the ABA biosynthesis genes may be common features of RNA virus infection. Increased levels of ABA in plants stressed by drought or infected with virus may eventually result in reduced growth, as found in the present study.

IAA can regulate diverse processes including growth, development and physiological metabolisms of plants (Kieffer et al., 2010; Swarup and Péret, 2012). Decreased IAA levels induced by drought stress have been reported in *Brassica napus* (Qaderi et al., 2006), *Phaseolus vulgaris* (Figueiredo et al., 2008), and *Cotinus coggygria* (Li et al., 2011). Reduced levels of IAA by virus infection were also found by Smith et al. (1968), Lockhart and Semancik (1970), Rao and Narasimham (1974), Rajagopal (1977), and Li et al. (2013). The results reported here were consistent with those mentioned above. The present study further found that co-stress by drought and virus resulted in much lower levels of IAA than single stress by either drought or virus, and down-regulation of expressions of IAA biosynthetic genes including TAR2, TAR3, TAR4, and YUC1 was associated with the decreased levels of IAA. Systemic virus infection was shown to influence the intercellular transport of the infected plants (Jameson and Clarke, 2002), which may prevent directional transport of auxin out of its source tissues, thus resulting in inhibition of shoot growth and root formation, as reported in the present study.

In conclusion, GLRaV-3 infection or drought stress reduced vegetative growth, affected physiological metabolisms, enhanced cell membrane damage and cell death, increased accumulation of H_2_O_2_ and ${\text{O}}_{2}^{\cdot -}$, Co-stresses by virus infection and drought cause much more deleterious effects, than either single stress, on the studied parameters. Analysis of total soluble protein, free proline, activities of antioxidant enzymes, MDA, levels of ABA and IAA and expressions of their biosynthetic genes provide insights into better understanding of the adverse effects of virus infection and drought, in single and in combination, on the grapevine *in vitro* plantlets. Results obtained in the present study address use of virus-free plants and good irrigation toward achieving sustainable development of the grapevine.

## Author contributions

ZC main work of all the experiments, data analysis and manuscript. WB phytohormone analysis and ROS gene expression analysis. XH the activity of the antioxidant enzymes YX design of the experiment and the financial support PL valuable discussion. AW the guide of the experiment and manuscript revision. QW design of the experiment, revision of the manuscript and the financial support.

### Conflict of interest statement

The authors declare that the research was conducted in the absence of any commercial or financial relationships that could be construed as a potential conflict of interest.

## Abbreviations

ABA: abscisic acid
BM: basal medium
CAT: catalase
DAB, 3: 3^*'*^-diaminobenzidine
DW: dry weight
EL: electrolytic leakage
FW: fresh weight
GLRaV-3: *Grapevine leafroll-associated virus-3*
IAA: indoleactic acid
MDA: Methane Dicarboxylic Aldehyde
MS: Murashige and Skoog medium
NBT: nitroblue tetrazolium
PCD: Programmed cell death
PEG: polyethylene glycol
POD: peroxidase
ROS: Reactive oxygen species
SOD: superoxide dismutase.
